# Unmasking Portal Colopathy: A Case of Rectal Bleeding in a Patient With Liver Cirrhosis Misdiagnosed as Ulcerative Colitis

**DOI:** 10.7759/cureus.78494

**Published:** 2025-02-04

**Authors:** Chanchal Kumar Ghosh, Aditi Sarkar, Sumona Islam, Nafizul Islam, Amit Bari

**Affiliations:** 1 Department of Gastroenterology, Bangabandhu Sheikh Mujib Medical University, Dhaka, BGD; 2 Department of Gastroenterology, Bangladesh Medical College Hospital, Dhaka, BGD; 3 Department of Nephrology, Kidney Foundation Hospital and Research Institute, Dhaka, BGD

**Keywords:** chronic hepatitis, cirrhosis, gastrointestinal hemorrhage, portal hypertension, portal hypertensive colopathy, ulcerative colitis

## Abstract

This report outlines the diagnostic journey of a 31-year-old female patient with chronic hepatitis B virus (HBV) infection who presented with acute rectal bleeding. Initially, she was misdiagnosed with ulcerative colitis at a primary care center, though the underlying cause of her symptoms was related to cirrhosis and its complication, portal hypertensive colopathy (PHC). At presentation, she revealed a history of ascites a few months before the onset of rectal bleeding, which had been managed with diuretics. Subsequent investigations led to a revised diagnosis of decompensated cirrhosis secondary to chronic HBV infection, with PHC identified as the cause of her rectal bleeding. This case highlights the diagnostic challenges faced when managing patients with coexisting cirrhosis and gastrointestinal bleeding, initially misdiagnosed as ulcerative colitis.

## Introduction

Portal hypertension occurs when the hepatic venous pressure gradient exceeds 5 mmHg [1]. This condition causes mucosal and hemodynamic changes throughout the gastrointestinal (GI) tract, leading to complications such as portal hypertensive gastropathy and portal hypertensive colopathy (PHC). Both can present with GI hemorrhage [2]. Patients with cirrhosis should undergo colonoscopy to assess for portal hypertension-related lesions in the colorectal region, as PHC is a significant cause of lower GI bleeding in cirrhotic patients [3]. This report discusses a case of PHC presenting with rectal bleeding, initially misdiagnosed as ulcerative colitis.

## Case presentation

A 31-year-old woman, a known hepatitis B surface antigen (HBsAg) carrier, was referred to our hospital as a case of ulcerative colitis from a primary care center. She denied any history of jaundice, hematemesis, melena, altered consciousness, or sleep disturbances and had no history of blood transfusions, surgeries, sexual promiscuity, or intravenous drug use.

The patient had a past history of ascites, diagnosed 11 months prior to the current presentation. Her ascites had been managed effectively with diuretics at that time. Six months later, she developed abrupt-onset acute rectal bleeding, occurring five to six times daily. The bleeding was bright red, occasionally mixed with stool, and not associated with urgency or tenesmus. She underwent colonoscopy and histopathology, and based on her clinical features, colonoscopic findings, and histopathological results, she was misdiagnosed with ulcerative colitis and was treated with mesalamine, corticosteroids, and thiopurines, with gradual dose adjustments. While she initially showed some improvement, she subsequently experienced recurrent ascites, fresh rectal bleeding, and melena.

At the presentation at our institute, the patient was found to be anemic, non-icteric, and had bilateral pedal pitting edema. Abdominal examination revealed ascites and mild splenomegaly. Laboratory findings showed anemia (Hb 9.6 g/dl), thrombocytopenia (80,000/µL), prolonged prothrombin time, and hypoalbuminemia (2.13 g/dl). She was HBsAg-positive, with undetectable hepatitis B virus (HBV) DNA. Anti HBc IgG was positive. Ascitic fluid analysis revealed a high serum-ascitic albumin gradient (SAAG), consistent with transudative ascites. Details of the investigation are in Table 1.

**Table 1:** Laboratory investigations Hb: hemoglobin; AST: aspartate aminotransferase; ALT: alanine aminotransferase; SAAG: serum-ascites albumin gradiant; HBsAg: hepatitis B surface antigen; HBeAg: hepatitis B virus e antigen; Anti-HBs: hepatitis B surface antibody; HBV: hepatitis B virus

Investigations	Patient value	Reference value
Hb (g/dl)	9.6	12-16
Platelet (/μL)	80,000	150,000-450,000
Serum creatinine (μmol/L)	70	55-97
AST (U/L)	43	10-34
ALT (U/L)	47	10-49
Prothrombin time (seconds)	16	12
Serum albumin (g/dl)	2.13	3.5-5
HBsAg	+	
HBeAg	_	
AntiHBs	_	
HBV DNA	undetectable	
SAAG (g/dl)	1.2	

**Figure 1:**
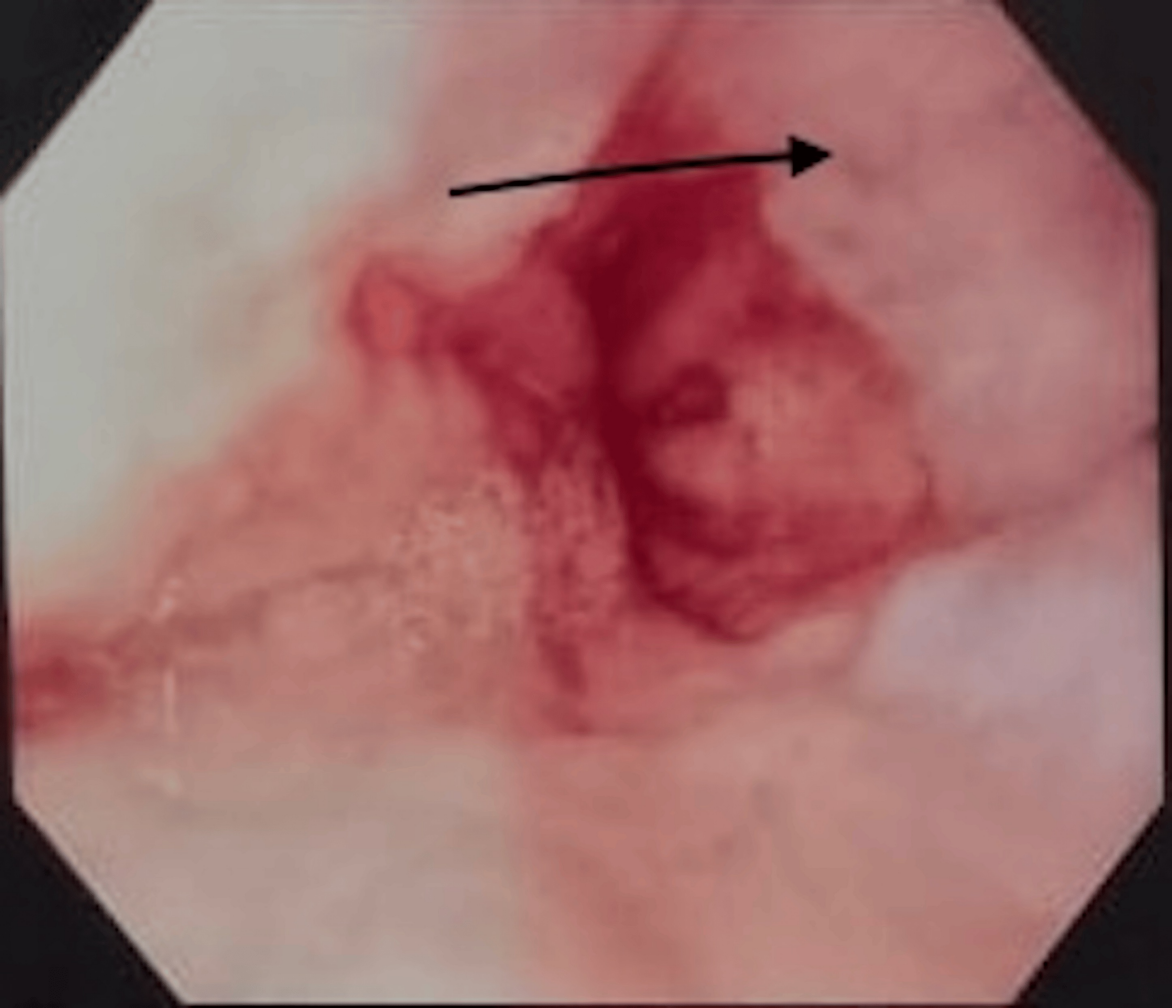
Endoscopy showing medium sized esophageal varices

**Figure 2:**
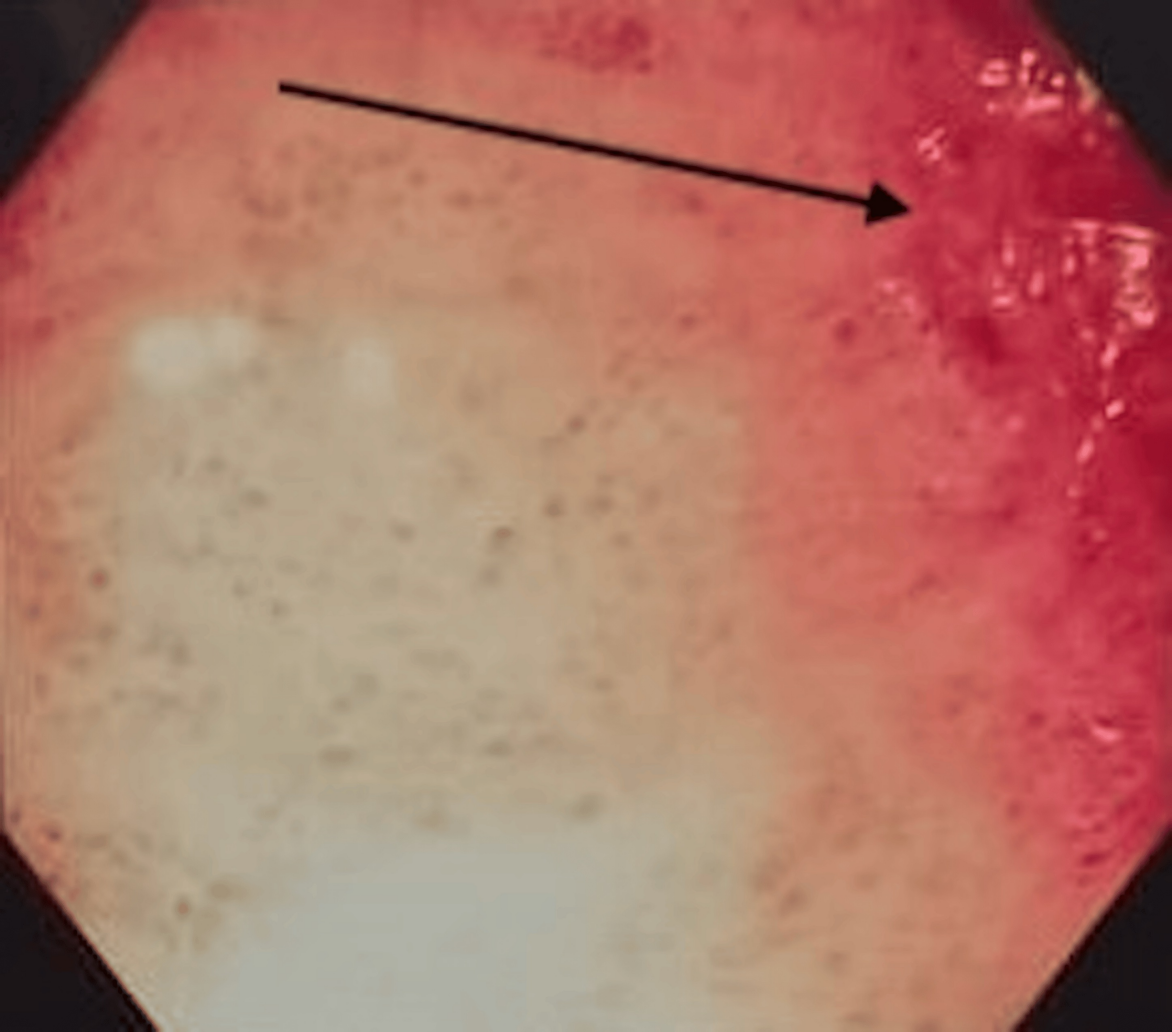
Endoscopy showing portal hypertensive gastropathy

**Figure 3:**
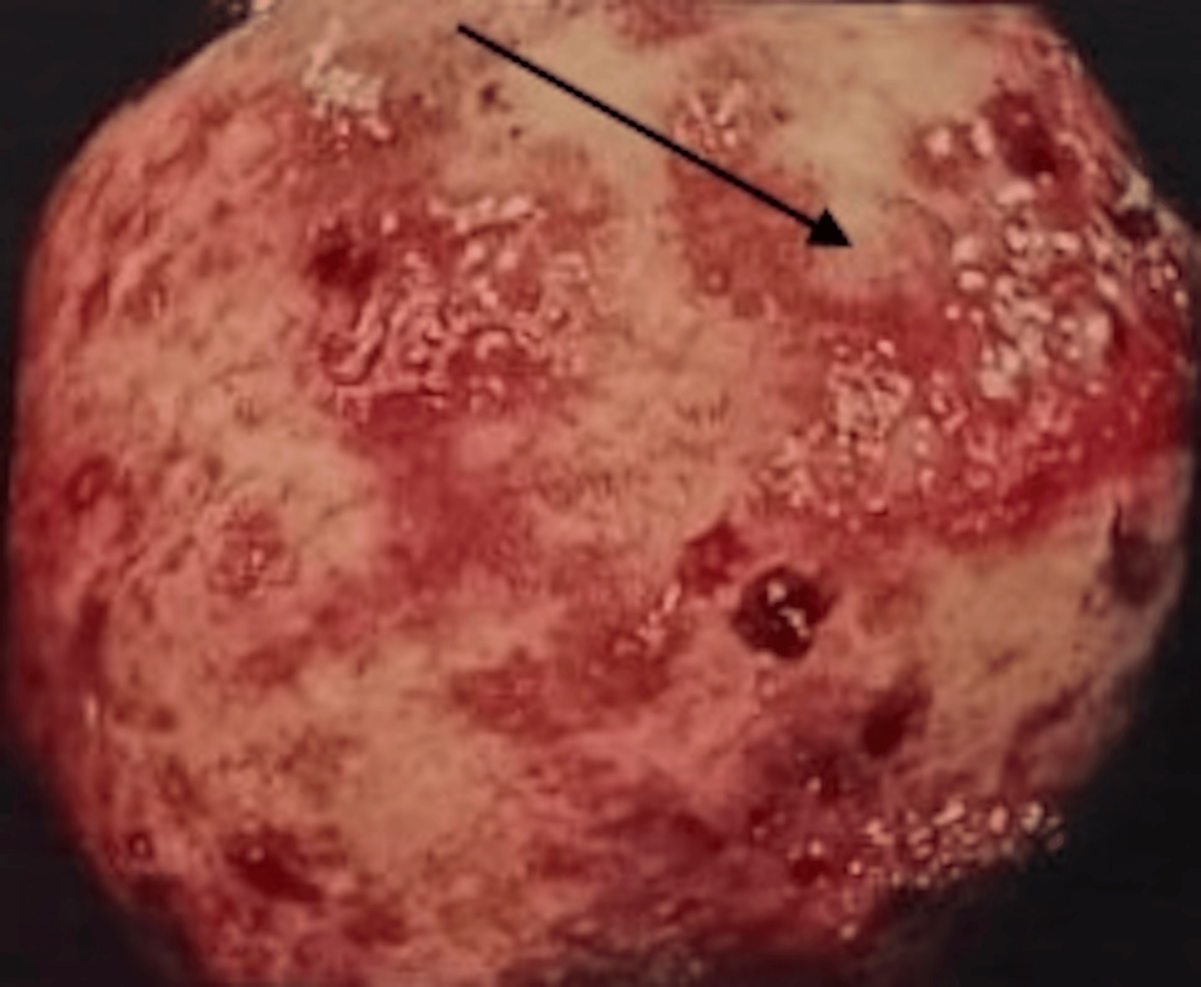
Mucosal erythema in spider like fashion in colon

Imaging studies demonstrated coarse hepatic parenchyma, mild splenomegaly, moderate ascites, and a dilated portal vein suggestive of portal hypertension. Fibroscan results indicated advanced fibrosis (F3). Endoscopy revealed medium-sized esophageal varices (Figure 1) and portal hypertensive gastropathy (Figure 2), while colonoscopy showed mucosal erythema in a spider-like pattern, consistent with PHC (Figure 3).

**Figure 4:**
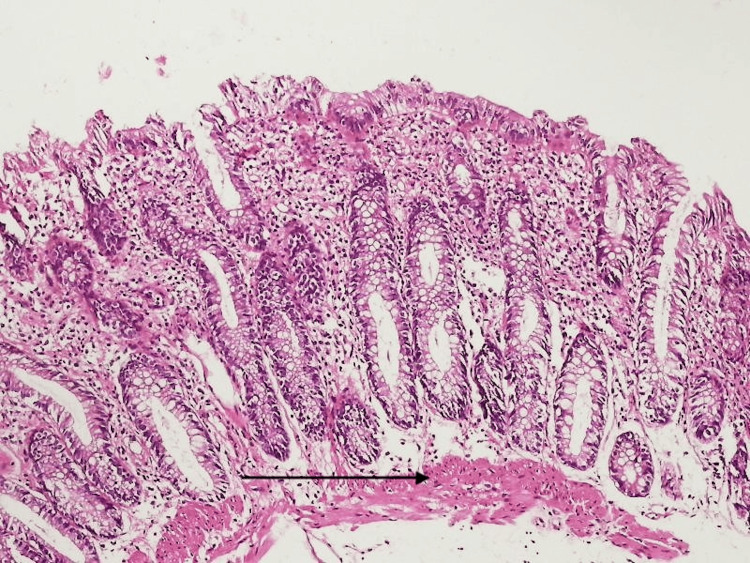
Histopathology of colon shows non-specific proctitis

Histopathology findings were non-specific, showing proctitis without evidence of ulcerative colitis (Figure 4).

The diagnosis was revised to decompensated cirrhosis due to chronic HBV infection, with PHC as the cause of the rectal bleeding.

The patient was treated with antiviral therapy, propranolol, spironolactone, furosemide, and iron supplementation. Mesalamine was discontinued after colonoscopy findings confirmed no loss of vascular pattern or ulceration in the colon. Despite adherence to her treatment plan, the patient required repeated hospitalizations for anemia and was transfused with blood and iron. During one hospitalization for profuse melena and rectal bleeding with features of shock, she was stabilized and treated with terlipressin, endoscopic variceal ligation (EVL), propranolol, and iron supplementation. At the four-month follow-up, her clinical condition was seen to have improved significantly, with no further episodes of GI bleeding or melena.

## Discussion

PHC is an underrecognized yet clinically significant complication of portal hypertension, often presenting with lower GI bleeding in cirrhotic patients. While it shares similarities with other portal hypertension-related complications, such as esophageal varices and portal hypertensive gastropathy, its diagnosis and management pose unique challenges [3,4]. PHC is characterized by mucosal and vascular changes in the colon caused by increased portal venous pressure, leading to vascular dilatation, mucosal edema, and friability. These changes make patients prone to rectal bleeding, which may mimic other GI conditions, such as inflammatory bowel disease (IBD) [4]. This overlap in clinical and endoscopic findings can complicate the diagnostic process, as demonstrated in this case.

This patient was initially misdiagnosed with ulcerative colitis based on her clinical presentation, colonoscopic findings, and histopathological analysis. Bright red rectal bleeding and mucosal erythema observed during colonoscopy were incorrectly attributed to IBD, which led to the initiation of inappropriate therapies, including corticosteroids, 5-aminosalicylates, and thiopurines. Although there was a transient improvement in her symptoms, the patient later experienced recurrent rectal bleeding and other features of decompensated cirrhosis such as ascites and melena. This clinical deterioration prompted further investigations, which ultimately revealed the underlying condition of PHC secondary to chronic HBV-related cirrhosis [4,5].

The diagnostic process highlights the importance of maintaining a high index of suspicion for PHC in patients with cirrhosis and lower GI bleeding. Colonoscopy remains a cornerstone in the diagnosis of PHC, with characteristic findings that include mucosal erythema, edema, vascular ectasia, and a spider-like vascular pattern. In contrast to IBD, PHC typically lacks chronic inflammatory changes, crypt distortion, or ulcerations on histopathology [4,6]. In this case, the patient’s biopsy showed non-specific proctitis without evidence of ulcerative colitis, which, when correlated with her advanced liver disease, supported the diagnosis of PHC.

PHC as a cause of GI bleeding is not as well-recognized as esophageal varices or portal hypertensive gastropathy. This lack of awareness often leads to misdiagnosis and delayed treatment, as seen in this case. Furthermore, the patient’s misdiagnosis and prolonged exposure to immunosuppressive therapy could have worsened her underlying liver condition and increased her vulnerability to infections and complications of decompensated cirrhosis. This case underscores the need for a multidisciplinary approach, including hepatologists, gastroenterologists, and pathologists, when evaluating cirrhotic patients with GI bleeding [7-10].

Management of PHC primarily focuses on addressing the underlying portal hypertension. Non-selective beta-blockers, such as propranolol, are often used to reduce portal pressure and the risk of recurrent bleeding. EVL and terlipressin may also play a role in managing acute bleeding episodes, as demonstrated in this patient. Long-term outcomes depend on the degree of portal hypertension and the success of interventions to control the underlying liver disease. For patients with decompensated cirrhosis, liver transplantation remains the definitive treatment [10,11].

This case also highlights the importance of a comprehensive clinical history and the integration of findings from various diagnostic modalities to arrive at the correct diagnosis. A detailed history of ascites preceding rectal bleeding could have raised earlier suspicion of an alternative diagnosis, potentially avoiding unnecessary treatments and delays in appropriate care. Additionally, the absence of extraintestinal features typically seen in IBD, such as arthropathy, uveitis, or skin manifestations, might have further guided clinicians away from an IBD diagnosis.

## Conclusions

PHC is an underdiagnosed cause of lower GI bleeding in patients with cirrhosis. This case underscores the importance of a thorough diagnostic workup, including colonoscopy, in cirrhotic patients presenting with GI symptoms. he diagnosis of cirrhosis was missed in this case when the patient came with ascites and this is a learning point. Timely diagnosis and appropriate management can improve outcomes and reduce the burden of unnecessary treatments.
